# Volatile-Mediated Effects Predominate in *Paraburkholderia phytofirmans* Growth Promotion and Salt Stress Tolerance of *Arabidopsis thaliana*

**DOI:** 10.3389/fmicb.2016.01838

**Published:** 2016-11-17

**Authors:** Thomas Ledger, Sandy Rojas, Tania Timmermann, Ignacio Pinedo, María J. Poupin, Tatiana Garrido, Pablo Richter, Javier Tamayo, Raúl Donoso

**Affiliations:** ^1^Laboratorio de Bioingeniería, Facultad de Ingeniería y Ciencias, Universidad Adolfo IbáñezSantiago, Chile; ^2^Center of Applied Ecology and SustainabilitySantiago, Chile; ^3^Millennium Nucleus Center for Plant Systems and Synthetic BiologySantiago, Chile; ^4^Departamento de Química Inorgánica y Analítica, Facultad de Ciencias Químicas y Farmacéuticas, Universidad de ChileSantiago, Chile

**Keywords:** plant growth promoting rhizobacteria (PGPR), *Paraburkholderia phytofirmans* PsJN, *Arabidopsis thaliana*, abiotic stress tolerance, ACC deaminase, volatile organic compounds (VOCs)

## Abstract

Abiotic stress has a growing impact on plant growth and agricultural activity worldwide. Specific plant growth promoting rhizobacteria have been reported to stimulate growth and tolerance to abiotic stress in plants, and molecular mechanisms like phytohormone synthesis and 1-aminocyclopropane-1-carboxylate deamination are usual candidates proposed to mediate these bacterial effects. *Paraburkholderia phytofirmans* PsJN is able to promote growth of several plant hosts, and improve their tolerance to chilling, drought and salinity. This work investigated bacterial determinants involved in PsJN stimulation of growth and salinity tolerance in *Arabidopsis thaliana*, showing bacteria enable plants to survive long-term salinity treatment, accumulating less sodium within leaf tissues relative to non-inoculated controls. Inactivation of specific bacterial genes encoding ACC deaminase, auxin catabolism, *N*-acyl-homoserine-lactone production, and flagellin synthesis showed these functions have little influence on bacterial induction of salinity tolerance. Volatile organic compound emission from strain PsJN was shown to reproduce the effects of direct bacterial inoculation of roots, increasing plant growth rate and tolerance to salinity evaluated both *in vitro* and in soil. Furthermore, early exposure to VOCs from *P. phytofirmans* was sufficient to stimulate long-term effects observed in *Arabidopsis* growth in the presence and absence of salinity. Organic compounds were analyzed in the headspace of PsJN cultures, showing production of 2-undecanone, 7-hexanol, 3-methylbutanol and dimethyl disulfide. Exposure of *A. thaliana* to different quantities of these molecules showed that they are able to influence growth in a wide range of added amounts. Exposure to a blend of the first three compounds was found to mimic the effects of PsJN on both general growth promotion and salinity tolerance. To our knowledge, this is the first report on volatile compound-mediated induction of plant abiotic stress tolerance by a *Paraburkholderia* species.

## Introduction

Plants have evolved diverse mechanisms to cope with and survive environmental abiotic stresses such as salinity, including an early response to the short-term impact of high sodium concentrations, which is characterized by a rapid growth arrest by inhibition of cell growth and division (Munns and Tester, 2008), and the adjustment of osmotic potential by the cellular accumulation of compatible solutes (Ismail et al., 2014). Although there is a great variety in the extent of salinity tolerance among diverse land plants, there is substantial evidence on the activation of additional cell protection mechanisms, like ROS detoxification (Gill and Tuteja, 2010), sodium exclusion (Roy et al., 2014) and its storage within vacuoles (Fan et al., 2014), to achieve tolerance to saline stress in glycophyte plants (Munns and Tester, 2008). This includes most food crops, as well as the model plant, *Arabidopsis thaliana* (Hauser and Horie, 2010; Zhang and Shi, 2013). Remarkably, it has been shown that a tolerance response can be improved in *A. thaliana* by external stimuli, improving the efficacy of osmotic adjustment and ion detoxification mechanisms (Jakab et al., 2005). These induced systemic tolerance (IST) events are probably mediated by the interplay of hormone signaling pathways within the plants, including salicylic acid (SA), ethylene and jasmonic acid-pathways (Cho et al., 2008). Currently, the environmental signals underlying induction of IST responses are not well understood. However, they represent attractive targets for enhancing productivity and growth of crop plants under field conditions (Farag et al., 2013).

Microorganisms play a key role providing conditions necessary for plant growth and development in a variety of environments (Lugtenberg and Kamilova, 2009; Vacheron et al., 2013; Liu and Zhang, 2015). Interestingly, it has been found that colonization of plants by certain specific plant growth promoting rhizobacteria (PGPR) can lead to enhanced resistance to abiotic challenges, such as water deficit (Naveed et al., 2014), salinity (Sziderics et al., 2007), adaptation to transplantation (Nowak and Shulaev, 2003), and chilling (Ait Barka et al., 2006). Over the last few years, several studies have reported the ability of isolated microorganisms to induce plant tolerance to salinity once they have been inoculated to seeds or young plantlets (reviewed in Yang et al., 2009; Dodd and Pérez-Alfocea, 2012; Shrivastava and Kumar, 2015), including a variety of hosts, like wheat (Nadeem et al., 2013; Singh et al., 2015), maize (Hamdia et al., 2004; Nadeem et al., 2009), cotton (Liu et al., 2013; Egamberdieva et al., 2015), tomato (Mayak et al., 2004; Ali et al., 2014), lettuce (Barassi et al., 2006; Kohler et al., 2009), sunflower (Shilev et al., 2010; Tewari and Arora, 2014) and *Arabidopsis* (Zhang et al., 2008; Kim et al., 2014; Sukweenadhi et al., 2015). Among the PGPR that have been demonstrated to play a role in salt stress tolerance induction, a wide diversity of bacteria is included, encompassing several members of the γ-proteobacteria class, specially within the genus *Pseudomonas* (Ahmad et al., 2013; Nadeem et al., 2013; Chang et al., 2014; Han et al., 2015), α-proteobacteria belonging to the *Azospirillum* genus (del Amor and Cuadra-Crespo, 2011; Nia et al., 2012; Sahoo et al., 2014), and β-proteobacteria like *Achromobacter* (Mayak et al., 2004) or *Paraburkholderia* (Talbi et al., 2013; Pinedo et al., 2015). Several examples have also been described for the phylum Firmicutes, with special emphasis on the genus *Bacillus* (Zhang et al., 2008; Kohler et al., 2009; Karlidag et al., 2013; Ramadoss et al., 2013; Han et al., 2015), and also some examples have been described within the Actinobacteria phylum (Sadeghi et al., 2012; Palaniyandi et al., 2014; Gond et al., 2015). Despite these numerous reports on induction of plant tolerance to salinity, relatively few studies have been able to identify microbial mechanisms involved in the induction of salt tolerance in their hosts. For many beneficial plant bacteria interactions in saline conditions, a role has been proposed for the deamination of the ethylene precursor 1-aminocyclopropane-1-carboxylate (ACC), to produce ammonia and α-ketobutyrate (Glick et al., 2007), as a bacterial modulator of growth and tolerance to saline stress in the host, which is supported by reports involving mainly *Pseudomonas* and *Enterobacter* species isolated for their ability to metabolize ACC (Mayak et al., 2004; Nadeem et al., 2007; Saravanakumar and Samiyappan, 2007; Ahmad et al., 2013; Chang et al., 2014). Other phytohormone-related bacterial functions, like the ability to modulate indoleacetic acid (IAA) levels, have also been proposed to play a role in plant salt stress tolerance induction (Shilev et al., 2010; Wu et al., 2012; Egamberdieva et al., 2015). However, it is not easy to rule out the contribution of additional putative growth promotion functions, like the production of siderophore compounds, which is frequently observed in bacterial isolates effective in amelioration of abiotic stress (Barriuso et al., 2008; Shilev et al., 2010; Tiwari et al., 2011; Ramadoss et al., 2013; Liu and Zhang, 2015; Singh et al., 2015; Lee et al., 2016). These may act as stimulating compounds by themselves, activating a tolerance response within the plant (Aznar and Dellagi, 2015). Additionally, production of bacterial exopolysaccharide (EPS) might be able to trigger an analogous response and even physically protect roots from osmotic stress by adhering to root surface and reducing ion absorption (Sandhya et al., 2009; Tewari and Arora, 2014). Finally, the emission of volatile organic compounds (VOCs) has been shown to be widespread in soil and rhizosphere bacteria, taking part in several types of biotic interactions (Effmert et al., 2012; Lemfack et al., 2014). Abiotic stress tolerance induction by VOC emission has been demonstrated for certain *Bacillus* species, like *Bacillus subtilis* GB03 (Zhang et al., 2008), a mechanism that may be shared by other PGPR bacteria, belonging to different phyla (Blom et al., 2011), including the γ-Proteobacterium *Pseudomonas simiae* AU (Vaishnav et al., 2015, 2016). So far, studies focused on characterization of phytostimulating VOCs have demonstrated that active emissions are usually produced as complex mixtures of different compounds, and these components vary greatly among tested PGPR strains, even when these are close phylogenetic relatives (Farag et al., 2006; Blom et al., 2011). In general terms, although reports of plant growth stimulation and concomitant stress tolerance induction by rhizosphere bacteria are abundant in literature, there are comparatively few instances where the specific contribution of bacterial determinants has been studied by inactivation of relevant genes, and detailed analysis of plant growth stimulation to reveal the actual functional significance of each candidate mechanism.

*Paraburkholderia phytofirmans* PsJN is a widely studied plant growth promoter, with outstanding abilities for colonization of different plant hosts, including tomato (Sharma and Nowak, 1998), potato (Frommel et al., 1991; Kurepin et al., 2015), grape (Compant et al., 2005; Trdá et al., 2014), ryegrass (Afzal et al., 2013) switchgrass (Kim et al., 2012), lupin (Kost et al., 2014), maize (Naveed et al., 2015), and *Arabidopsis* (Poupin et al., 2013). A versatile and effective model for PGPR-mediated plant stimulation, strain PsJN has been demonstrated to promote plant growth and improve the host tolerance to several biotic and abiotic stress conditions (Sessitsch et al., 2005). In *Vitis vinifera* L. cv Chardonnay, PsJN colonizes roots, stem and leaves tissue (Compant et al., 2005), and induces systemic protection of the host plant against pathogen attack (Compant et al., 2005, 2008), an effect that can also be observed in *A. thaliana* (Timmermann et al., unpublished). Additionally, *P. phytofirmans* PsJN colonization is also able to enhance the tolerance of inoculated plants to water deficiency and drought-derived damage (Naveed et al., 2014; Wang et al., 2016), to protect from transplantation (Nowak and Shulaev, 2003), osmotic and chilling stress (Ait Barka et al., 2006; Fernandez et al., 2012), and from freezing temperatures in *Arabidopsis* (Su et al., 2015). The strain has a functional ACC deaminase (Sun et al., 2009; Zúñiga et al., 2013), as well as the ability to synthesize and degrade IAA (Compant et al., 2005; Donoso et al., 2016), and AHL mediated quorum sensing has been shown to regulate plant tissue colonization (Trognitz et al., 2008; Zúñiga et al., 2013). On the other hand, the ability to emit VOCs has been collectively reported for several *Burkholderia* and *Paraburkholderia* species, including *P. phytofirmans* (Blom et al., 2011; Weisskopf and Bailly, 2013), but the specific contribution of this potential mechanism to the overall growth promotion effects of the individual bacteria, and a possible role in stress tolerance induction, has not been explored so far.

We have recently described the ability of *P. phytofirmans* PsJN to enhance salt tolerance of *A. thaliana*, focusing on metabolic and transcriptional changes in the plant, related to stress-perception and stress-responsive pathways (Pinedo et al., 2015). That study showed that PsJN inoculation increased tolerance to salt treatment in short and long-term exposure, suggesting a priming (IST) effect mediated by bacterial inoculation, which involves modulation of the transcriptional response of the plant in the presence of stress. However, the bacterial effectors involved in these effects are yet unclear, and it is currently uncertain whether a stable colonization of the host is required throughout the plant life cycle to achieve an effective phytostimulation. In this work, we have aimed to further characterize the extent of PsJN-mediated tolerance in *A. thaliana*, by exploring if protection against the lethal effects of salinity, and a better sodium management, can be induced by the bacterium. We have especially endeavored to identify the key molecular mechanisms of *P. phytofirmans* involved in plant growth stimulation and salt-stress tolerance. For this, we have evaluated plant development under salt-stress using bacterial mutants in key growth promotion functions, which enabled us to analyze the relevance of direct phytohormone modulation and colonization on tolerance induction, and we studied the contribution of bacterial VOC emissions to the stimulant effects of strain PsJN. Our observations highlight the importance of this last phytostimulation mechanism over other possible PGPR functions, not only for induction of salinity tolerance in the host plant, but for general plant growth stimulation by beneficial *Paraburkholderia* strains.

## Materials and Methods

### Bacterial Strains, Growth Conditions, and Plant Inoculation

*Paraburkholderia phytofirmans* PsJN was originally obtained from the laboratory of Dr. A. Sessitsch (AIT, Austria). *Azospirillum brasilense* Sp7 was acquired from DSMZ. Wild type Sp7, PsJN, and four PsJN mutants were routinely grown at 30°C in Dorn mineral salts medium (Dorn et al., 1974) containing 10 mM fructose, and supplemented with kanamycin (Km, 50 μg ml^-1^), whenever required (see below). Bacterial inocula were grown in a rotary shaker (150 rpm) up to mid-exponential phase (O. D. 600 = 0.6). Cell suspensions were collected, adjusted to approximately 10^8^ colony forming units (CFU ml^-1^) and diluted in agar media, just prior to solidification, at specific bacterial concentrations for plant inoculation in gnotobiotic systems. To assess the effect of heat-inactivated bacteria, an inoculum suspension was heated at 95°C for 20 min prior to the final dilution in agar medium (HK-PsJN inoculum). Mortality was routinely confirmed by plate counting.

PsJN-*acdS*, PsJN-*iacC*, and PsJN-*bpI.1* mutants were constructed as described previously (Zúñiga et al., 2013). Briefly, each open reading frame (loci Bphyt_5397; Bphyt_2156; Bphyt_0126, respectively), was disrupted by insertional recombination using a suicidal kanamycin resistance plasmid containing a cloned internal segment of the target gene reading frame, to get one homologous recombination event and thus generate two truncated copies of the selected gene, obtaining *P. phytofirmans* PsJN respective mutants, which were selected on LB agar containing 50 μg ml^-1^ Km. Changes in the acyl homoserine lactone profile of mutant *bpI.1* were verified (Zúñiga et al., unpublished results), together with the loss of the ability to catabolize IAA (Donoso et al., 2016) in PsJN-*iacC*, and the ACC deaminase function in the PsJN-*acdS* mutant, respectively. This last strain completely lost activity, measured as the production of α-ketobutyrate from ACC [58 nmol α-ketobutyrate (mg protein)^-1^ min^-1^ in the wild type strain] (Penrose and Glick, 2003), as well as the ability to use ACC as a sole nitrogen source (data not shown). The *fliA* gene mutant, was obtained by using primer pairs fliAmutFW (5′- GCACAAGGTGGAGCAGAATC -3′), and fliAmutRV (5′- TACAGCGACATCAGCAGCTT -3′). The PCR gene product was cloned using the pCR2.1-TOPO system (Invitrogen, Carlsbad, CA. USA), to generate suicidal plasmid pCR2.1*fliA*. This plasmid was then introduced into *P. phytofirmans* PsJN by electroporation, to get one recombination event disruption of the *fliA* gene, and potential recombinants were selected in Luria Bertani (LB) agar medium containing kanamycin. Correct insertion within the *fliA* ORF was confirmed by PCR and sequencing. Swimming motility in Dorn 0.3% agar medium supplemented with 10 mM fructose was completely abolished in the mutant strain (data not shown).

### *Arabidopsis thaliana* Growth and Exposure to Salinity *In soil*

*Arabidopsis thaliana* seeds were obtained from the ABRC. These were surface sterilized with 2.5% sodium hypochlorite (a 1:1 mixture of commercial laundry bleach with water) containing 0.1% Tween 20, rinsed three times with sterile water, and maintained at 4°C for 7 days to synchronize germination. Square Petri dishes were prepared with half-strength Murashige-Skoog medium (MS^1^/_2_) (Murashige and Skoog, 1962) in 0.8% agar and inoculated or not with PsJN or its mutants, and surface sterilized seeds were sown to allow germination. For irrigation experiments with saline solutions in soil, germinated seeds were incubated under a 16:8 (long day) light:dark cycle at 20–22°C, and transferred to 1:1 peat:vermiculite substrate 11 days after sowing (DAS). Both inoculated and non-inoculated plants were irrigated every 48 h with sterile water. One day a week, plants received irrigation with a sterile solution of NaCl/CaCl_2_ 100 mM/10 mM, 200 mM/20 mM, 300 mM/30 mM, or with sterile water (untreated controls). Soil salinity was expected to increase through time in this experimental system, due to the cumulative effect of successive NaCl/CaCl_2_ irrigations, which was confirmed by soil conductivity measurements, indicating that saline irrigation increased soil conductivity from 0.4 ± 0.05 dS/m at the day of transplant, to 3.3 ± 0.11 dS/m, 8 ± 0.16 dS/m, and 12.3 ± 0.21 dS/m, for 100 mM/10 mM, 200 mM/20 mM, and 300 mM/30 mM NaCl/CaCl_2_, respectively, at day 36. Rosette growth was registered photographically at 5–6 days intervals and total rosette area was estimated every week using Adobe Photoshop Cs3 software (Adobe Systems Incorporated, San Jose, CA, USA), and fresh (FW) and dry weight (DW) quantified at the end of the experiment with an analytical balance (Shimadzu Corporation, Japan).

### *Arabidopsis thaliana* Growth and Exposure to Salinity *In vitro*

To measure the sort-term effect of saline shock in PsJN inoculated and non-inoculated *A. thaliana*, plants were transferred at 11 DAS to MS^1^/_2_ supplemented with salt concentrations ranging from 0 mM NaCl/0 mM CaCl_2_ to 250 mM NaCl/25 mM CaCl_2_. Different growth parameters (root length and rosette diameter) and leaf senescence signs were evaluated during 14 days after transfer. For experiments involving *in vitro* exposure to salt, plants were maintained in their original square Petri dishes for 21 DAS, at different NaCl/CaCl_2_ concentrations, and final FW and DW were determined, as well as growth parameters. Leaf/rosette area measurements of plants *in soil* or *in vitro* experiments were calculated using Adobe Photoshop as stated above. Statistical analyses of plant growth parameters and leaf senescence were performed using one-way analysis of variance (ANOVA). Tukey’s honestly significant difference (*P* < 0.05) test was used to make comparisons among different treatments. Homogeneity of variances and normality tests were performed using the MiniTab Statistical Software (MiniTab Incorporated, State College, PA, USA).

### Sodium Concentrations in Plant Tissues

Inoculated and non-inoculated salt-stressed plants were harvested at different times from soil or *in vitro* experiments for sodium extraction and quantification, which was performed as in Zhang et al. (2008). For soil experiments, plant rosettes were collected, weighed, and treated separately. For *in vitro* grown plants, roots and rosettes of five plants (one plate), within each experimental group were collected and pooled. Roots and rosettes were weighed separately and rinsed with deionized water. Plant tissue was dried at 60°C for 2 days, and dry weight was registered for each pool of separated tissue. All material was subsequently extracted with 3 ml of 100% HNO_3_ overnight, followed by incubation at 90 to 100°C for 1 h. Aqueous Na^+^ in this final solution was determined by atomic absorption spectrophotometry (Model 3110; PerkinElmer Instruments, Norwalk, CT, USA), and normalized relative to dry weight.

### Growth Stimulation by Emission of Volatile Organic Compounds from *Paraburkholderia phytofirmans* PsJN

*Arabidopsis thaliana* seeds ecotype Col-0 were sterilized and sown on MS^1^/_2_ medium with 0.8% agar in one half of the plate. On the other half with Dorn mineral salts medium containing 10 mM fructose as the sole carbon and energy source and 1.5% agar, 1 × 10^6^ CFU of each strain (HK-PsJN, PsJN, and GB03) were inoculated over a membrane (pore size of 0,22 μm) and the plates were sealed with parafilm. Twenty days after inoculation FW and chlorophyll content measurements were performed, as described by Porra et al. (1989). Total chlorophyll was extracted from leaves of *A. thaliana* using N,N-9-dimethylformamide for 24 h at 4°C in the dark, and chlorophyll a and b concentrations were measured simultaneously by spectrophotometry. To test for significant differences in response variables, one-way ANOVA were performed, using Shapiro–Wilk test for normality, and Bartlett tests for homogeneity of variances. Statistical analyses were carried out using R software. When differences in the means were significant, a Tukey’s HSD test was performed.

To further evaluate the effects of bacterial volatile emissions on plant growth, quantifying root growth, seeds were exposed to bacteria using a sealed dual agar plate system, where *P. phytofirmans* PsJN, a mutant strain, or a heat killed inoculum, was homogenously inoculated in MS^1^/_2_ 0.8% agar medium in one square Petri dish, and *A. thaliana* seeds were sown in a separate MS^1^/_2_ plate. Both plates were then placed in front of each other, and held together using parafilm. Non-exposed plantlets were incubated in front of sterile MS^1^/_2_ agar medium. To assess the contribution of CO_2_ accumulation due to parafilm sealing, control experiments were performed to measure plant growth parameters in both sealed and non-sealed dual plate systems. To evaluate long term effects of volatile exposure, volatile treated and non-treated plants were transferred to soil after 11 DAS, and plant growth was monitored until day 40. Alternatively, plantlets were transferred to fresh MS^1^/_2_ agar supplemented or not with 150 mM NaCl/15 mM CaCl_2_, to test for the effects of early exposition to volatiles on the development of *A. thaliana* to salt tolerance. Plant growth parameters and leaf senescence were measured as described above. To evaluate the effect of individual volatile compounds on plant growth and salinity tolerance, the dual plate system described above was modified by introducing an open sterile 0.6 ml eppendorf tube, attached to the center of the plate in front of *A. thaliana* seeds, in the absence of bacterial inoculums. Different amounts, ranging from 100 ng to 1 mg of each of the selected volatile compounds 2-UN, 3-MB, 1-HEP, or DMDS, were placed into the attached tube prior to sealing the plates together. As volatilization of the compounds was visually confirmed, addition of the indicated amounts was repeated periodically (once every 5 days) for the rest of the stimulation period (until 14 DAS) to avoid eventual loss of the respective volatiles throughout incubation time. This was done by opening the system and re-sealing after application. After the stimulation period, plantlets were transferred to fresh medium in the presence or absence of salt, and growth parameters were determined at the times indicated above.

### Detection of Volatiles Emitted by *Paraburkholderia phytofirmans* PsJN

For detection of bacterial volatiles, strain PsJN was placed in 20 ml headspace tubes, homogenously inoculated in MS^1^/_2_ at a density of 10^6^ CFU ml^-1^, prior to airtight sealing. Alternatively, bacteria were placed on the surface of 10 ml of solid 0.8% agar supplemented with MS^1^/_2_ or Kings B medium. After 72 h incubation at 30°C to allow for bacterial growth, 100 μl samples were taken through the septum of each tube using a GC syringe, and directly loaded into a Hewlett Packard Gas Chromatographer (injection temperature set at 160°C), equipped with an HP-5 column 30 m × 320 μm × 0.25 μm, Agilent Technologies (5%- phenyl, 95%-methylpolysiloxane), and a flame ionization detector (FID) detection system (detector temperature 200°C). Runs were performed in a splitless mode, with N_2_ as make-up (20 ml min^-1^) and carrier gas (2.3 ml min^-1^). A temperature ramp was applied to detect as many volatile signals as possible, starting at 70°C for 2 min, then programmed at a rate of 6°C min^-1^ to 71°C for 2 min, and finally ramped at a rate of 120°C min^-1^ to 155°C for 2 min. Headspace volumes of tight sealed control tubes with MS^1^/_2_ of Kings B medium in the absence of bacteria, were injected as negative controls. Under these conditions, four clear signals could be detected from PsJN-inoculated Kings B medium, and three of these appeared also in MS^1^/_2_ inoculated tubes. To confirm the identity of the volatile compounds detected, the headspace compounds of PsJN-inoculated tubes were concentrated using a solid phase micro-extraction (SPME) (50/30 μm divinylbenzen/carboxenTM/polydimethylsiloxane (PDMS)) 2 cm stableflex/ss fiber exposed to volatile emission within headspace tubes for 30 min at 30°C, and run in a Shimadzu GC equipment coupled to MS, under the following conditions: Injector: 270°C; detector: 210°C; and column oven 40°C for 2 min, then programmed at a rate of 5°C min^-1^ to 110°C, and finally ramped at a rate of 10°C min^-1^ to 280°C. Separation was achieved using a 60 m × 0.25 mm × 0.25 μm (5%-diphenyl-95% polymethylsiloxane) DB-5MS column (Agilent Technologies), and carrier gas was He, set to a flow velocity of 1 ml min^-1^. Obtained profiles unequivocally identified the detected signals as 2-UN; 3-MB; 1-HEP; and DMDS by comparison with the NIST library. This was further confirmed in both the GC-MS and GC-FID systems by comparison with analytical standards of each separate compound, obtained from Sigma-Aldrich (Milwaukee, WI, USA).

## Results

### Bacterial Features Favoring Host Colonization and Alteration of Phytohormone Levels Do Not Influence Stimulation of *A. thaliana* Salinity Tolerance by *P. phytofirmans* PsJN

Previous results from our group suggest that early PsJN inoculation is sufficient to enhance tolerance to salinity in *A. thaliana*, rather than just increasing growth rate regardless of saline stress (Pinedo et al., 2015). However, an estimation of salinity-induced mortality, senescence and tissue damage is necessary to ascertain if sodium exclusion or tissue tolerance mechanisms are being activated in the plants (Roy et al., 2014), and to determine if salt toxicity is effectively reduced by bacterial inoculation. Supplementary Figure S1 summarizes the most relevant differences in rosette growth among PsJN inoculated and N. I. *A. thaliana* plants growing under different saline stress conditions in soil, showing that inoculated plants do not only survive, but continue to grow in the presence of salt, in contrast with N. I. controls, and that they can retain over 60% of water content when grown under saline irrigation, while plants without inoculum retained only 40% under the same conditions (Supplementary Table S1). The salinity tolerance index (DW_salt_/DW_control_), measured for inoculated plants was 0.74, compared to 0.51 for N. I. plants, and sodium concentrations within leaves reached 32 ± 8.9 mg Na^+^/g of dry tissue in N. I. controls irrigated with 200/20 mM of NaCl/CaCl_2_, while PsJN-inoculated plants reached only 19.7 ± 5.2 mg Na^+^/g of dry tissue. This is consistent with a reduced sodium accumulation in rosette tissues when PsJN-inoculated plants are exposed to salinity *in vitro*, relative to accumulation within N. I. controls (Supplementary Table S2), while several other growth parameters are modified by bacterial inoculation under the same salinity conditions (Supplementary Figures S2 and S3 ).

To explore the role of bacterial functions putatively involved in both plant growth promotion and induction of plant stress tolerance, the effects of *P. phytofirmans* PsJN mutants were compared to the wild type, regarding stimulation of *A. thaliana* tolerance and growth under salt stress *in vitro*, as described in the previous section. Two of the selected mutants carried a recombinational insertion in genes related to bacterial modulation of phytohormone levels: Mutant strain PsJN-*AcdS* underwent inactivation of the ACC deaminase gene (*acdS*) which only has one chromosomal copy in *P. phytofirmans* PsJN. This inactivation completely abolished ACC deaminase activity, and also the ability of the strain to use ACC as a sole nitrogen source for growth (Supplementary Figure S4). On the other hand, mutant strain PsJN-*IacC*, carried an insertion in a specific aromatic ring hydroxylating dioxygenase gene (*iacC*), which is involved in the catabolism of the auxin indole-3-acetic acid (IAA) by *P. phytofirmans*. *A. thaliana* seeds were germinated *in vitro* in the presence of each of these mutants, the wild type *P. phytofirmans*, HK-PsJN, or N. I. controls. Then, 11 DAS, plants were transferred to fresh MS^1^/_2_ medium supplemented with 0 (control) or 150/15 mM NaCl/CaCl_2_, and incubated for 2 weeks prior to evaluation of plant growth parameters.

**FIGURE 1:**
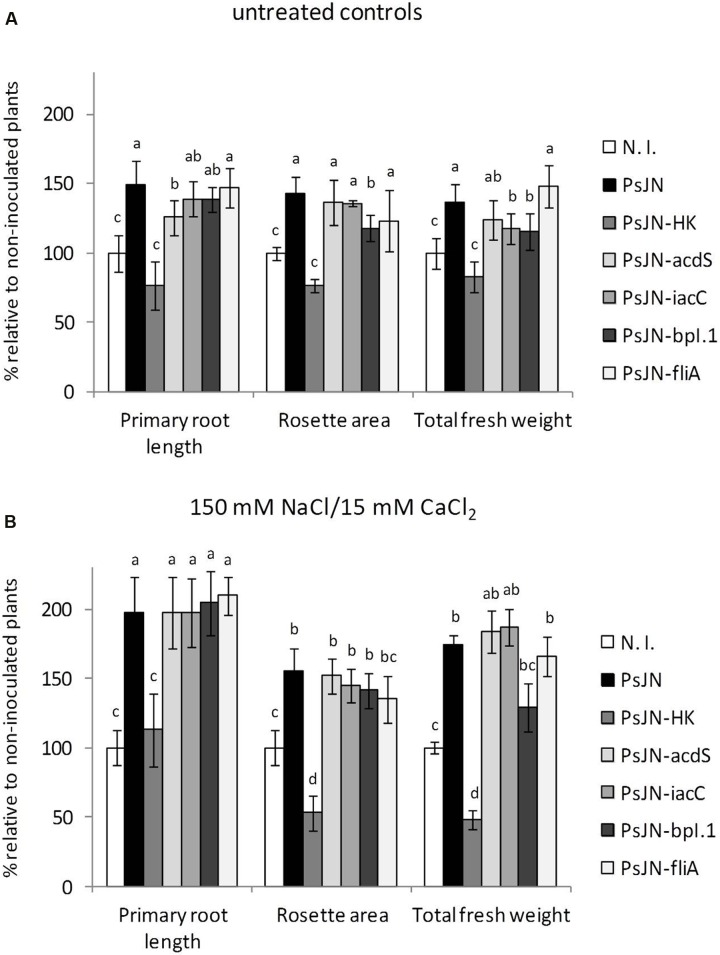
**Effect of *Paraburkholderia phytofirmans* PsJN-derived mutants on growth stimulation of *Arabidopsis thaliana* plants.** Root growth, rosette area and fresh weight of *A. thaliana* col-0 grown in gnotobiotic *in vitro* cultures using half strength MS agar medium inoculated with 1 × 10^4^ CFU/ml of *P. phytofirmans* (PsJN), a heat-killed PsJN inoculum (HK-PsJN), the PsJN-derived mutants (*acdS*, *iacC, bpI.1*, or *fliA*), or non-inoculated medium (N. I.). Plants were transferred at 11 days after sowing (DAS) to fresh MS^1^/_2_ agar with no added NaCl or CaCl_2_ (untreated controls) **(A)**, or MS^1^/_2_ agar with 150/15 mM NaCl/CaCl_2_ **(B)**. Growth parameters were registered at 21 DAS. Bars show mean percentage values relative to N. I. plants, and the error bars indicate standard deviations from experiments with 30 plants analyzed for each bacterium and salt treatment. Different letters indicate statistically significant differences among bacterial treatments within each salt concentration for each measured parameter (One way ANOVA Tukey’s HSD tests; *p* < 0.05).

As seen in **Figure 1**, inactivation of either *acdS* or *iaaC* did not result in a significant reduction of the ability of strain PsJN to stimulate *A. thaliana* salt stress tolerance, although certain significant differences could be detected with the wild type for growth stimulation of non-stressed plants (**Figure 1A**). Consequently, mutant strains of *P. phytofirmans* in colonization related functions were also included in the analysis, in order to test their influence on PsJN-mediated salinity tolerance in *A. thaliana*. When PsJN-*BpI.1* inoculated plants were challenged with 150/15 mM NaCl/CaCl_2_, a concentration producing the highest differences among wild type PsJN inoculated and N. I. salt stressed plants (Pinedo et al., 2015), the effect of the mutant displayed no significant differences to that of the wild type strain (**Figure 1B**). Finally, a mutant was produced with an inactivated version of gene *FliA*, which encodes for a main regulator protein controlling flagellar assembly, to investigate if flagella can work out as bacterial elicitors of IST in *A. thaliana*. PsJN-*FliA* proved unable to perform swimming motility toward fructose in soft agar (Supplementary Figure S5). As shown in **Figure 1B**, this last mutant strain showed identical growth promotion effects to those of the wild type PsJN, both in control and saline conditions. These results suggest that microbe-associated molecular patterns, colonization and/or modulation of phytohormone levels do not play a fundamental role in salt tolerance induction in *A. thaliana*.

### *Paraburkholderia phytofirmans* PsJN Promotes Growth of *A. thaliana* and Induces Saline Stress Tolerance by Emission of Volatile Organic Compounds

**FIGURE 2:**
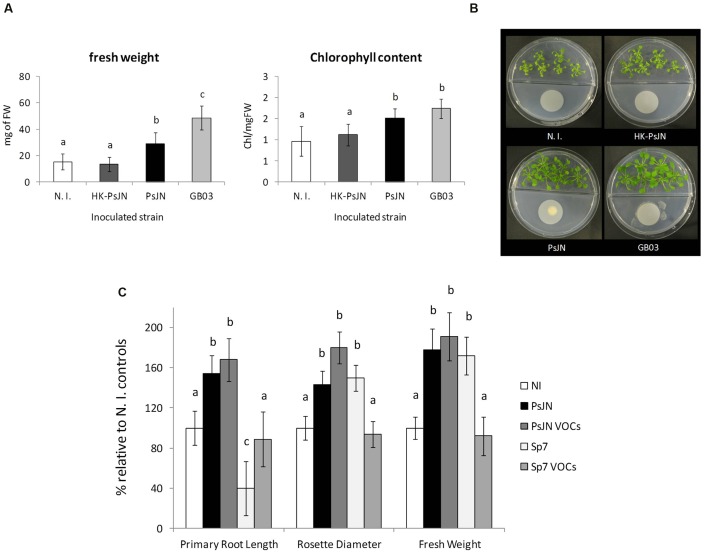
**Bacterial stimulation of *A. thaliana* growth mediated by volatile organic compounds (VOC) from *P. phytofirmans* PsJN.** The effects of bacterial emission of volatile compounds was tested using divided petri dishes with *A. thaliana* col-0 plants sown in half strength MS medium agar on one side, and minimal medium for bacterial growth on the other. 1 × 10^6^ CFU of the corresponding bacterial inoculum (N. I.; PsJN; HK-PsJN; or *Bacillus* sp. strain GB03) was added on top of a 0.022 μm filter. Total fresh weight and chlorophyll content per mg of fresh tissue were measured at 21 DAS. Columns show mean values, while error bars indicate standard deviations for 30 plants (fresh weight) or 6 plants (chlorophyll content) (**A**, left and center panels). Different letters indicate statistically significant differences among bacterial treatments (One-way ANOVA, *p* < 0.05). Representative photographs of plates are presented for each bacteria treatment **(B)**. Root growth, rosette diameter and fresh weight of *A. thaliana* **(C)** directly inoculated with 1 × 10^4^ CFU/ml of strain PsJN (PsJN), 1 × 10^4^ CFU/ml of *A. brasilense* strain Sp7 (Sp7), or co-incubated separately with each of these strains in dual plate systems (PsJN VOCs or Sp7 VOCs, respectively). Growth parameters were registered at 21 DAS. Bars show mean percentage values relative to N. I. plants, and the error bars indicate standard deviations from experiments with 24 plants analyzed for each bacterium and salt treatment. Different letters indicate statistically significant differences among bacterial treatments within each salt concentration for each measured parameter (One way ANOVA Tukey’s HSD tests; *p* < 0.05).

Since production of VOCs has been shown to induce plant growth promotion in several members of the phylum Firmicutes, and important members of the phylum Proteobacteria (Liu and Zhang, 2015), preliminary assays were performed using *P. phytofirmans* PsJN to assess growth promotion of *A. thaliana* without direct contact with the host. Standard divided plate assays were carried out to explore plant growth stimulation by strain PsJN added to sterile cellulose filters and placed on MS^1^/_2_ agar directly opposite to *Arabidopsis* seeds. Fresh weight and chlorophyll content of volatile-treated plants in the absence of salt were measured. **Figure 2** shows the effects of strain PsJN incubated separately from the host, compared to the effects of the well-characterized volatile compound producer *B. subtilis* GB03, confirming the ability of *P. phytofirmans* to stimulate growth of *A. thaliana* through this mechanism. Inoculum density effects on seedling exposure to volatile emissions from strain PsJN (Supplementary Figure S6), were explored using a dual plate assay approach, that allows quantitative monitoring of root growth. This was achieved by sowing *A. thaliana* seeds in square Petri dishes and coupling these front-to-front with MS^1^/_2_ agar square dishes homogenously inoculated with strain PsJN. Under these conditions, inoculated bacteria were able to maintain their abundance and viability for at least 2 weeks (an initial inoculum of 1 × 10^6^ CFU ml^-1^ allowed an average recovery of 5.8 × 10^5^ ± 3.2 × 10^5^ CFU ml^-1^ after 2 weeks), but did not show substantial growth due to the lack of an abundant carbon source in MS^1^/_2_ medium. Different bacterial inoculum concentrations were assayed in this system to explore the optimal microbial abundance for volatile-mediated promotion of *A. thaliana* growth. These experiments suggested no growth promotion, and even a mildly inhibitory effect, was produced when bacteria reached 1 × 10^8^ CFU/ml of agar medium, which is consistent with previous data using direct inoculation of roots with this number of bacteria (Poupin et al., 2013). However, inoculum concentrations of 1 × 10^4^ and 1 × 10^6^ were both able to promote plant growth in a similar fashion (Supplementary Figure S6). As a general rule, further experiments to analyze the effects of PsJN volatile emission on *A. thaliana* were performed using this dual plate system, and an inoculum concentration of 1 × 10^6^ CFU/ml of agar in front of the plants. The contact-independent effects of strain PsJN contrasted with those of the PGPR *A. brasilense* (**Figure 2**). This species was selected because it has been shown to increase the growth of different plant hosts, including *A. thaliana* (Dubrovsky et al., 1998; Fibach-Paldi et al., 2012), but it does so by a mechanism involving bacterial production of IAA, and direct contact between bacteria and plant roots (Dobbelaere et al., 1999; Spaepen et al., 2014). Here, direct inoculation of plant roots with Sp7 produced the expected increase in plant weight, and general modification of the root system architecture (shorter primary roots, but abundant lateral roots). However, when bacteria and plants were inoculated separately in our dual plate system (Sp7 VOCs), no significant increase in plant growth relative to non-inoculated control systems was observed (**Figure 2C**). In order to explore if the observed effects could arise from specific VOC-mediated stimulation of plant growth, or merely by accumulation of CO_2_ due to the use of a sealed system (Kai et al., 2016), control experiments were performed to compare the effects obtained in our sealed setting with a non-sealed dual plate system, which showed similar stimulation effects (Supplementary Figure S7).

**FIGURE 3:**
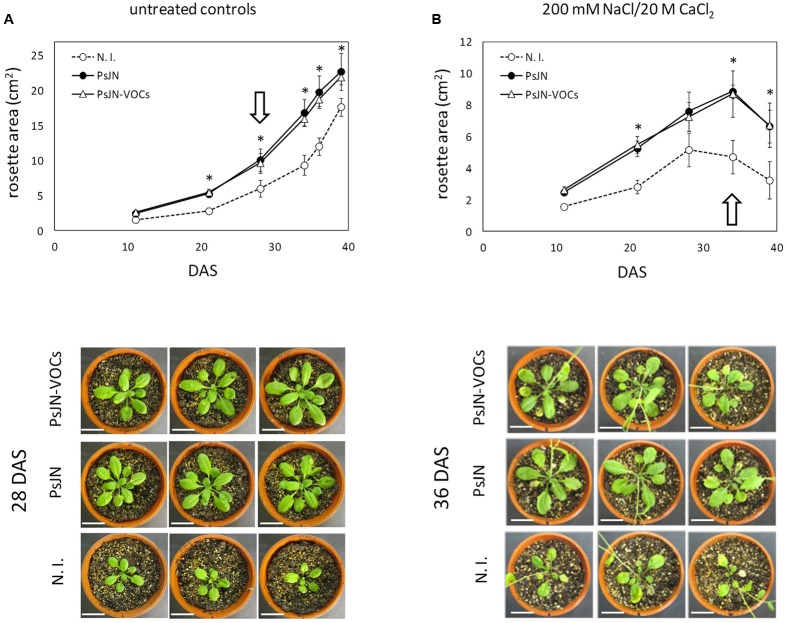
**Long-term effects of exposure to volatile emission from *P. phytofirmans.*** *Arabidopsis* seeds were exposed to PsJN volatiles (PsJN-VOCs), directly inoculated with the bacterium (PsJN), or non-inoculated (N. I.) in the dual plate system for 11 DAS, and plantlets were transferred to soil. Transferred plants were irrigated three times a week. Untreated controls received only standard irrigation water **(A)**, while treated plants received 2 irrigations with standard water and a third containing 200 mM of NaCl and 20 mM of CaCl_2_ **(B)**. Images of 10 representative plants taken at different times, and rosette area was measured for each treatment. Asterisks indicate statistically significant differences among PsJN and N. I: plants within each measured time point, which were explored using One-Way ANOVA Tukey’s HSD tests; *p* < 0.05. Images of representative plants are shown below each treatment, representing time points (DAS) with the highest differences in rosette area for untreated controls and salt irrigated plants (highlighted by white arrows).

**FIGURE 4:**
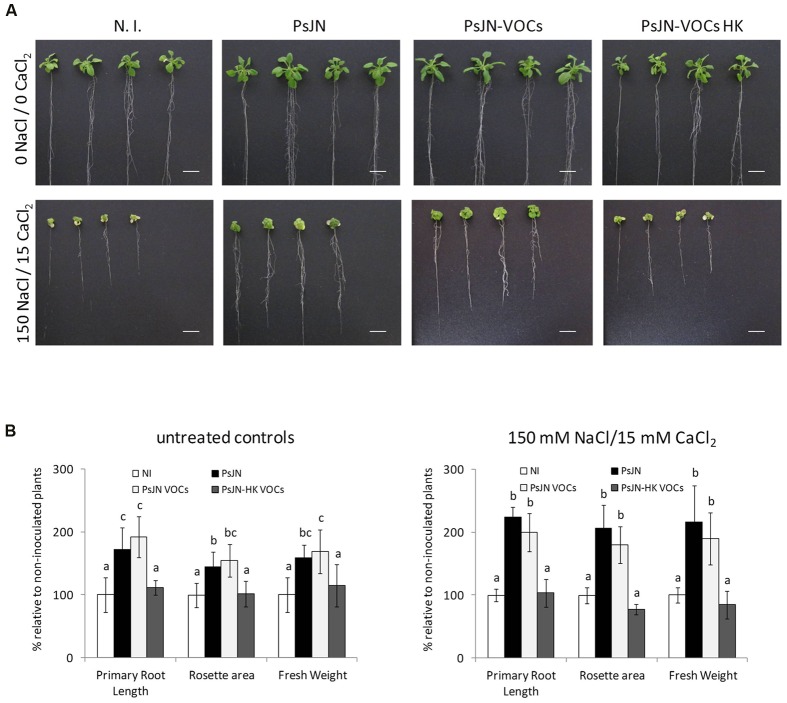
**Volatile organic compounds-mediated stimulation of *A. thaliana* tolerance to salinity.** Root growth, rosette diameter and fresh weight were measured for *A. thaliana* col-0 plants inoculated with *P. phytofirmans* (PsJN), non-inoculated (N.I), or exposed to volatile emissions from live PsJN (PsJN-VOCs) or a heat-killed inoculum (HK-PsJN-VOCs) (see Materials and Methods). Plants were transferred at 11 DAS to fresh MS^1^/_2_ agar with no added NaCl or CaCl_2_ (0 NaCl/0 CaCl_2_) as untreated controls (**A**, upper row; **B** left panel), or MS^1^/_2_ agar with 150/15 mM NaCl/CaCl_2_ (**A**, lower row; **B** right panel). Growth parameters were registered at 21 DAS. Bars show mean percentage values relative to N. I. plants, and the error bars indicate standard deviations from experiments with 30 plants analyzed for each bacterium and salt treatment. Different letters indicate statistically significant differences among bacterial treatments within each salt concentration for each measured parameter (One way ANOVA Tukey’s HSD tests; *p* < 0.05).

Long-term effects of volatile treatment during the 1st days of *Arabidopsis* growth were studied by transplanting 11 days old plantlets to peat:vermiculite soil substrate, and measuring growth parameters under standard culture and irrigation conditions. As can be seen in **Figure 3A**, early stimulation through volatile compound treatment (plants without contact with strain PsJN) allowed plants to grow at a similar rate than those directly inoculated with strain PsJN, showing that volatile treatment alone is able to reproduce an essential phytostimulation property of *P. phytofirmans*, and that exposure to these emissions during the first 11 days of plant development is sufficient to accelerate growth throughout the entire plant life-cycle. Furthermore, when plants exposed to PsJN volatile emissions were transferred to soil and irrigated with 200/20 mM NaCl/CaCl_2_, following the same regime described in Supplementary Figure S1, they exhibited similar tolerance levels to those directly in contact with the strain (**Figure 3B**), suggesting that volatile emissions by *P. phytofirmans* may be also sufficient to explain stress tolerance induction in *A. thaliana*, even though salinity stress was applied at a much later developmental stage than volatile treatment. Representative images of plants undergoing different inoculum and salinity conditions highlight the similarities among PsJN-inoculated and volatile-treated plants. To assess the effects of PsJN when a saline shock was applied *in vitro*, the effects of an 11 day co-incubation of *Arabidopsis* seeds with strain PsJN at 1 × 10^6^ CFU/ml were registered for plants that were subsequently transferred to fresh MS^1^/_2_ medium in the presence or absence of 150/15 mM NaCl/CaCl_2_ (**Figure 4**), and compared to the effect of direct inoculation of the plants with PsJN. These results showed that exposure to *P. phytofirmans* VOCs during the first 11 DAS was enough to stimulate plant growth to the same levels of directly inoculated plants, while the response of *A. thaliana* to saline treatment was not different among the two types of bacterial treatment. The volatile-mediated effect of strain PsJN could not be reproduced by inoculation of heat-killed bacteria to agar in front of *A. thaliana* seeds at a concentration equivalent to 1 × 10^6^ CFU/ml, showing that volatiles must be emitted by metabolically active bacteria (**Figures 4A,B**).

### Identification of VOCs Emitted by *P. phytofirmans* and Their Effect on *A. thaliana* Growth and Tolerance to Saline Stress

To identify VOC putatively responsible for the beneficial effects of strain PsJN, the headspace of *P. phytofirmans* cultures grown in MS^1^/_2_ (containing 10 mM fructose) and Kings B media in hermetically sealed tubes were analyzed by gas chromatography with a FID, to discriminate relevant signals. Specific selected samples were run through a gas chromatographer coupled to a mass spectrometer detector to allow identification of eluted peaks. Then, candidate compounds were compared with commercial standards. This strategy allowed identification of four major compounds in the headspace of strain PsJN: 2-undecanone (2-UN); 1-heptanol (1-HL); 3-methyl-butanol (3-MB); and dimethyl disuphide (DMDS) (Supplementary Figure S8). Each of the identified compounds was found to be emitted by PsJN at concentrations in the range of 0.05–10 μg per ml of air within headspace tubes, depending on the growth medium. Concentrations in tubes containing MS^1^/_2_ were approximately 0.1, 0.08, and 0.05 μg per ml for 3-MB, 1-HP, and 2-UN, respectively, compared to 8.0, 7.0, and 4.5 μg per ml in Kings B. The DMDS signal, on the other hand, was consistently absent from all MS^1^/_2_ samples (data not shown), and could only be observed when bacteria grew in Kings B medium, rising up to 10 μg per ml of air.

**FIGURE 5:**
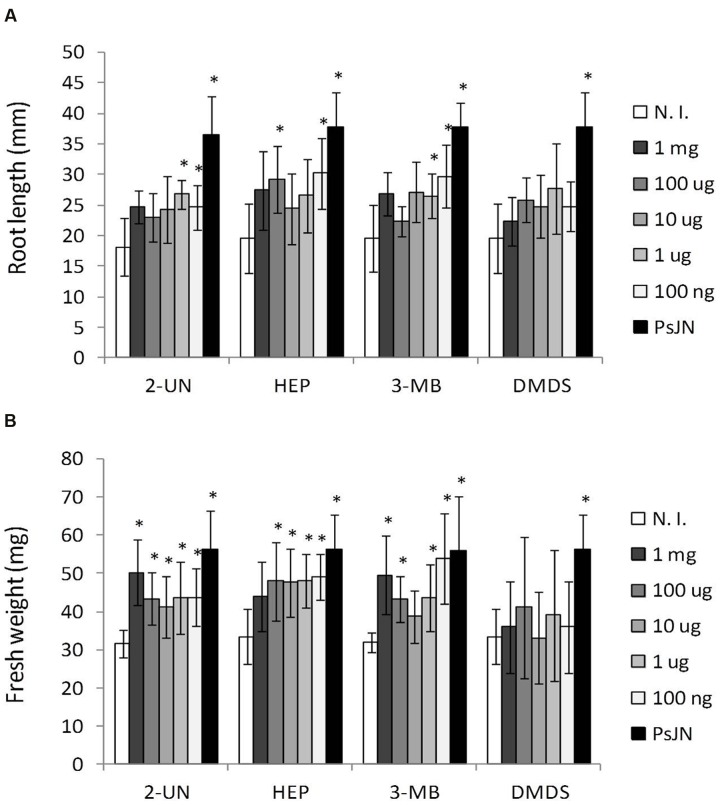
***Arabidopsis thaliana* growth stimulation by different amounts of individual volatile compounds emitted by *P. phytofirmans* PsJN.** Root growth **(A)** and fresh weight **(B)** were determined for *A. thaliana* col-0 grown *in vitro* MS^1^/_2_ agar medium without bacterial inoculum, and in the presence of different amounts of volatile compounds added per plate. Tested compounds were 2-undecanone (2-UN); 1-heptanol (1-HP); 3-methyl-butanol (3-MB); or dimethyl disulphide (DMDS) at the indicated amounts. Growth parameters were registered at 21 DAS. Columns show mean values, and the error bars indicate standard deviations from experiments with 24 plants analyzed for each compound concentration. Asterisks indicate statistically significant differences from the control (N. I.) treatment within each compound (One way ANOVA Tukey’s HSD tests; *p* < 0.05).

**FIGURE 6:**
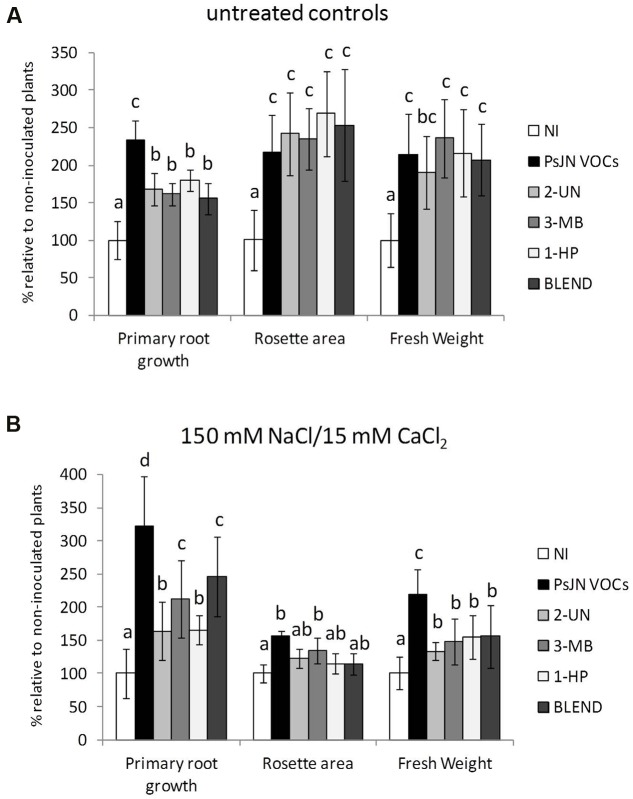
**Stimulation of *A. thaliana* growth and salt tolerance by individual volatile compounds and a volatile blend emitted by *P. phytofirmans* PsJN.** Root growth, rosette area and fresh weight were determined for *A. thaliana* col-0 grown *in vitro* on MS^1^/_2_ agar medium without bacterial inoculum, placed in front of an independent MS^1^/_2_ agar plate inoculated with 1 × 10^6^ CFU/ml of *P. phytofirmans* (PsJN-VOCs), non-inoculated MS^1^/_2_ agar (N. I.), or in the presence of different amounts of volatile compounds added per plate. Tested compounds were 100 ng of 2-undecanone (2-UN); 1-heptanol (1-HP); 3-methyl-butanol (3-MB); or a 1:1:1 blend of the three compounds (BLEND). Plants were transferred at 14 DAS to fresh MS^1^/_2_ agar with no added NaCl or CaCl_2_ (untreated controls) **(A)**, or MS^1^/_2_ agar with 150/15 mM NaCl/CaCl_2_ **(B)**. Growth parameters were registered at 21 DAS. Columns show mean values relative to N. I. controls, and the error bars indicate standard deviations from experiments with 24 plants analyzed for each compound treatment. Asterisks indicate statistically significant differences from the control (N. I.) treatment within each compound (One way ANOVA Tukey’s HSD tests; *p* < 0.05).

Using the *in vitro* dual plate stimulation method, described above, *A. thaliana* seeds were placed in MS^1^/_2_ in front of sterile medium (N. I. controls), PsJN-inoculated medium, or plates supplemented by addition of 2-UN, 1-HL, 3-MB, or DMDS at different amounts, ranging from 100 ng to 1 mg of the added compound per 100 ml of headspace volume (1 ng–10 μg per ml), contained in the assay system. These additions were repeated every 5 days of incubation, to compensate for the loss of volatiles throughout incubation time, and plants were harvested at 21 DAS, to test for growth promotion induced by each amount of supplemented bacterial volatile. Consistently with reported amounts found in the headspace of PsJN cultures, growth promoting amounts of individual volatiles range from 100 ng to 100 μg per plate, while higher values did not result in significant growth promotion effects in the plants (**Figure 5**). Finally, three volatile compounds, 2-UN, 1-HL, and 3-MB, were selected to test for stimulation of saline stress tolerance in plants. As previous experiments had determined production of these three compounds to be in the 0.1–0.05 μg per ml range in MS^1^/_2_, added amounts for this experiment should not exceed 5–2.5 μg in total for a 50 ml plate, in order to avoid testing concentrations higher than those that can be produced by bacteria under the experimental conditions. Thus, a low amount of 100 ng of each volatile was added periodically to separate plates of *Arabidopsis* seedlings in the dual plate system. Alternatively, a 1:1:1 blend of the three compounds was also added to plants during the first 14 DAS. Then, these were transferred to fresh medium in the presence or absence of 150/15 mM of NaCl/CaCl_2_, without further volatile-mediated stimulation, and incubated until 21 DAS. As shown in **Figure 6**, each of the identified volatiles is able to induce a partial level of plant growth promotion in *A. thaliana*, when added separately, or in the tripartite blend, in the absence of salt. This was especially significant in the case of rosette growth and total fresh weight stimulation, while the observed increases in root growth were lower than those produced by the actual volatile emissions of strain PsJN (PsJN VOCs) (**Figure 6A**). Regarding growth in the presence of salt, addition of separate compounds produced intermediate effects compared to those of PsJN emissions. However, some of these proved significantly different to non-stimulated control plants, as in the case of addition of 3-MB and 2-UN for stimulation of rosette growth, and the effect of these two compounds and the volatile blend on root growth and increase in total fresh weight under salinity stress conditions (**Figure 6B**). Altogether, these results show that PsJN-mediated effects on plant growth stimulation and induction of salinity tolerance can be reproduced, to different extents, by specific stimulation with *P. phytofirmans*-derived VOC, partially mimicking the effect of direct inoculation of the live bacterium to *A. thaliana* plants.

## Discussion

Bacterial determinants of growth promotion and salinity tolerance in *A. thaliana* in response to PsJN inoculation have been studied in this work, based on previous results showing that short- and long-term changes in the response of inoculated plants are able to enhance their growth in the presence of salt (Pinedo et al., 2015). Here, we have explored the onset of salinity tolerance conferred by *P. phytofirmans*, and the extent of long-term induction of plant growth and stress-survival by inoculation of strain PsJN, quantifying germination, growth parameters, sodium accumulation and water management in inoculated *A. thaliana* plants exposed to increasing concentrations of salt in irrigation water, and comparing these to N. I. control plants. The salinity tolerance index of inoculated plants under saline conditions was found to be similar to that of higher salt-tolerant ecotypes of *A. thaliana*, like Ws and Ler (Jha et al., 2010). Moreover, sodium levels measured in whole rosettes of stressed plants in soil showed a 40% lower amount of Na^+^ per gram of dry weight in PsJN-inoculated plants relative to N. I. controls, suggesting an increase of sodium exclusion capacity. This could be enough to explain relevant phenotype differences among the experimental groups, in terms of ion toxicity during long-term exposure to stress (Shi et al., 2002; Tester and Davenport, 2003). As PsJN was shown to influence *A. thaliana* tolerance to both long and short-term saline stress conditions, we used this simpler short-term model to study the role of specific bacterial functions on plant tolerance induction.

Several works have attempted to identify the main bacterial mechanisms involved in plant growth promotion and phytostimulation in *P. phytofirmans*, suggesting an involvement of IAA production (Naveed et al., 2015), acyl-homoserine lactone signaling (Trognitz et al., 2008; Zúñiga et al., 2013), ACC-deamination (Sun et al., 2009), and/or nicotinic acid mononucleotide metabolism (Wang et al., 2006). However, though phytohormone signaling is influenced by *P. phytofirmans* inoculation of different plant hosts, like potato (Kurepin et al., 2015), grapevine (Bordiec et al., 2011) and *Arabidopsis* (Poupin et al., 2013), it has not been successfully established if such changes are directly produced by bacterial synthesis/degradation of plant hormones, if they are induced by the plant in response to PsJN colonization, or even if they are indirect regulatory consequences of bacteria-triggered physiological changes, activated through different mechanisms. In terms of phytohormone signaling, strain PsJN has been reported to produce IAA from tryptophan (Mitter et al., 2013), which is further degraded by an aromatic cleavage catabolism pathway encoded by the *iac/cat* genes (Donoso et al., 2016). Additionally, *P. phytofirmans* ACC deaminase has been reported to enhance elongation of canola roots in the gnotobiotic pouch assay (Sun et al., 2009), while an *acdS* mutant that only differed from the wild type *P. phytofirmans* in ACC-deaminase displayed a significant reduction in root elongation of *A. thaliana* (Zúñiga et al., 2013). However, although the results of this work cannot fully exclude a role for bacterial IAA metabolism and ACC deamination in salinity tolerance-induction, they strongly suggest that these functions have a minor influence in the response of *Arabidopsis* under salt stress. This is not necessarily contradictory to the reported evidences mentioned above since, in the case of IAA metabolism, bacterial modulation of IAA levels would be expected to alter the response of the plants as long as the relevant bacterial pathways are induced and sufficiently active *in planta*, with sufficient numbers of colonizing bacteria to ensure an effect, and properly tuned to plant metabolism under the selected experimental conditions (Spaepen et al., 2007). When such requirements are not adequately met, null or undesirable effects in plant growth have been observed in other systems (Xie et al., 1996; Persello-Cartieaux et al., 2003). On the other hand, a case for the involvement of ACC deamination as the main bacterial modulator of growth and tolerance to saline stress in plants is supported by examples involving AcdS mutants of *Pseudomonas* species (Cheng et al., 2012; Ali et al., 2014; Han et al., 2015) but, for the most part of the reports in literature, the actual extent of the contribution of this bacterial enzyme to salinity tolerance induction in the host is not explored in detail, and has been mostly inferred from the existence of a functional AcdS in active bacterial isolates (Onofre-Lemus et al., 2009; Ahmad et al., 2013; Chang et al., 2014; Singh et al., 2015). However, it is worth noting that, in several cases, high ACC deaminase activity levels in isolates do not correlate with a better performance in salinity tolerance induction (Zheng et al., 2008; Tank and Saraf, 2010; Tiwari et al., 2011; Liu et al., 2013; Mapelli et al., 2013; Ramadoss et al., 2013), or even growth promotion activity in general (Dey et al., 2004; Long et al., 2008; Bruto et al., 2014). Furthermore, it has been observed that, in order to be effective, ACC deamination within plant tissues must deal with feedback regulation of ethylene biosynthesis (Yang and Hoffman, 1984), that will stimulate ethylene production when ACC levels are low (Vacheron et al., 2013). In the case of *P. phytofirmans*-mediated stimulation of *A. thaliana*, our results show that the influence of ACC deamination is restricted to root growth elongation and architecture changes in unstressed plants.

For *P. phytofirmans*-mediated phytostimulation, tissue colonization numbers appear to modulate the plant responses to bacterial inoculation, resulting in growth promotion or growth inhibition, depending on bacterial concentrations in rhizosphere and within plant tissues (Poupin et al., 2013; Zúñiga et al., 2013), which suggests that an unbalanced amount of bacteria entering in contact with the roots can be stressful to the plants. Accordingly, a purified flagellin fragment (*flg*22 peptide) from PsJN has been observed to reduce *A. thaliana* growth, when added exogenously (Trdá et al., 2014), while treatment with heat killed-bacteria does not benefit plant growth and stress tolerance (Pinedo et al., 2015 and this work), but induces stress pathways, that are not activated by live PsJN (Poupin et al., 2013). In the present study, a reduction of growth promotion and salt stress tolerance induction was observed for the *bpI.1* acyl-homoserine lactone signaling mutant of strain PsJN (**Figure 1**), which may be explained by its regulatory influence on a relevant growth promotion trait, or simply by its capacity to modulate plant colonization, derived from its role in rhizosphere competence, swimming motility and adherence to root tissues (Zúñiga et al., 2013). On the other hand, the PsJN-*fliA* strain, defective in the flagellum synthesis regulator FliA, induced salinity tolerance of *A. thaliana* just like the wild type PsJN, which suggests that colonization alone is not enough to induce salt tolerance and growth promotion and may be rather an additional source of biotic stress for the plants when not restricted below certain specific levels. In the context of PsJN-induced salinity tolerance in *A. thaliana*, colonization may be only required to achieve sufficient bacterial numbers in the proximity of the plant, since no evidence was found of a direct dependence of plant-bacteria contact on stimulation of growth and salinity tolerance. This does not imply that other promotion mechanisms may not be active and relevant in the absence of salt stress, while contact-independent growth promotion traits, such as volatile-mediated phytostimulation, may be less active in different environmental contexts as well.

As an intriguing alternative to contact-dependent plant growth promotion mechanisms, organic volatile compound production by PGPRs has been shown to be responsible for growth stimulation and stress tolerance induction in a variety of plant hosts (Liu and Zhang, 2015). It was first described for *Bacillus* stimulation of ISR in *Arabidopsis* (Ryu et al., 2003), and has since been shown to be relevant for the growth promotion of several plants (Farag et al., 2013), and to induce different physiological changes in the host, to modify gene expression patterns, and phytohormone levels, including cytokinin, IAA and abscisic acid, and even induce catabolism of certain bacterial volatiles (Oikawa and Lerdau, 2013). *Paenibacillus polymyxa* and *B. subtilis* have been thoroughly described to induce a stress tolerance response and priming in *A. thaliana*, providing protection against abiotic stress (Yang et al., 2009) and pathogen infection (Farag et al., 2006). It has been conclusively demonstrated that *P. polymyxa* exerts its protective actions through volatile emission of the C-13 compound tridecane (Lee et al., 2012), while *Bacillus* produces a complex mixture of compounds (Farag et al., 2006), that are able to activate diverse signaling pathways in the plant host (Zhang et al., 2008).

So far, phytostimulation by VOC production has been thoroughly studied mainly in Firmicutes and certain specific Proteobacteria, like *Pseudomonas chlororaphis*, but only a few molecules being unequivocally identified as plant growth modulators (Chung et al., 2016). However, the ability to produce volatile compounds currently appears to be widely distributed among rhizosphere bacteria, and is probably present in most plant associated species (Blom et al., 2011; Kanchiswamy et al., 2015). Though by far less understood, increasing evidence has been found for their involvement in many plant-bacteria interactions, including rhizosphere colonizers of the *Pseudomonas*, *Serratia* and *Burkholderia* genera, that produce a whole set of novel volatile compounds, different from those identified in *Bacilli* (Schulz and Dickschat, 2007). Descriptive studies have confirmed that volatile compounds in microbial emissions occur as a complex mixture of low-molecular weight lipophilic compounds, including alkanes, long chain alcohols, ketones, and sulfur compounds derived from different biosynthetic pathways (Schulz and Dickschat, 2007; Kanchiswamy et al., 2015). Despite the fact that several members of the *Burkholderia* (now *Paraburkholderia*) genus have been found to display plant growth promoting abilities (Compant et al., 2008; Suárez-Moreno et al., 2012), there are only a few detailed studies of the effects produced by volatiles emitted by members of the genus, and most are specially focused on antagonistic effects by *B. tropica* and *B. ambifaria* (Groenhagen et al., 2013; Tenorio-Salgado et al., 2013; Weisskopf and Bailly, 2013). Blom et al. (2011) showed the production of a wide variety of VOCs from different Proteobacteria strains, encompassing several species within the order Burkholderiales, including *P. phytofirmans*. However, volatile mixtures were shown to depend strongly on growth medium, and even vary considerably among closely related species. Moreover, no correlation could be observed among those volatile compounds and plants beneficial effects mediated by the bacteria included in their study and/or their reported type of interaction with plants, except for the case of *B. pyrrocinia* (Blom et al., 2011). We have analyzed VOCs production in *P. phytofirmans* PsJN by co-incubation of *A. thaliana* with bacteria homogenously inoculated in MS^1^/_2_ agar in sealed dual plate systems, showing that the resulting effects on plant growth are different from those of PGP bacteria that do not stimulate growth through volatile emission, like *A. brasilense*, which could not induce significant changes in plant size or weight when separately co-incubated with *A. thaliana*. In order to further explore the potential influence of CO_2_ in our sealed plates (Kai et al., 2016), we have assessed the effects of PsJN on plant growth in non-sealed systems, as recommended by Piechulla and Schnitzler (2016). The results of those experiments (Supplementary Figure S7) support the idea that PsJN-induced changes are not produced by CO_2_ accumulation.

We have also shown specific accumulation of the compounds 2-UN, 3-MB, 1-HL, and DMDS, in the headspace of cultures grown in MS^1^/_2_ medium, under conditions analogous to plant growth experiments, and we have confirmed their presence by comparison of retention times (Supplementary Figure S8) and MS profiles with those of analytical standards. We have explored the biological function of each compound added separately to *A. thaliana* cultures in different amounts (**Figure 5**). This suggests that most of them can produce an effect in a wide range of added quantities, with detectable influence on plant growth even at the lowest tested amounts (100 ng), which is roughly 50–100 times less that the amounts that could be detected in stationary phase PsJN cultures in sealed headspace tubes. On the other hand, some specific effects, like the increase in root length, were less pronounced when much higher amounts (1 mg) were tested. A similar observation was made by Blom et al. (2011) regarding addition of indole to *A. thaliana*, which promotes growth when tested in different amounts (1 ng–10 μg), but inhibits growth at 1 mg, while the study of the effects of different concentrations of volatile compounds of *Paenibacillus* on the elicitation of ISR, have shown that sometimes lower doses are required for a beneficial response (Lee et al., 2012). Our results support the potential of 2-UN, 3-MB, and 1-HL for direct growth promotion stimulation on *A. thaliana* (**Figure 6**), and suggest the involvement of one or more of these compounds in plant growth promotion and stress tolerance induction by *P. phytofirmans*. Furthermore, this study suggests that volatile-mediated effects on plant growth by strain PsJN are triggered at an early stage of plant growth, and are then able to influence the complete life cycle of the host, which is consistent with the onset of an IST response, as has been shown for *B. subtilis* stimulated tolerance (Zhang et al., 2008). Intriguingly, the volatile emissions from *P. phytofirmans* seem to be much less complex in terms of the diversity of its constituent compounds than that of *B. subtilis* strain GB03, as we could only detect the four indicated compounds, compared to more than 30 that have been found in *Bacillus* emissions (Farag et al., 2006; Lee et al., 2012) using a similar method, which could explain some of the differences in their growth promotion effects. However, it is noteworthy that PsJN volatile-mediated effects are able to influence plant growth in the long-term even though exposition takes place only during the first 11 days of growth, reaching similar tolerance levels to those of inoculated plants (**Figure 3**), an interesting feature which demands further study.

In summary, we have found that *P. phytofirmans* PsJN is able to promote growth and induce tolerance to salinity in *A. thaliana* mainly through the emission of volatile compounds, and that the contribution of bacterial phytohormone synthesis pathways is only minor. This is especially significant since, even though growth promotion by *Paraburkholderia* volatile emission has been reported before (Blom et al., 2011; Weisskopf and Bailly, 2013), relevant literature has failed to consider it as a possible mechanism underlying in the multiple plant growth promotion effects produced by *P. phytofirmans* PsJN, one of the most studied PGPR belonging to the β-proteobacteria (Suárez-Moreno et al., 2012; Mitter et al., 2013; Hardoim et al., 2015). The results of this work highlight the importance of the use of bacterial mutants in simple, well characterized, plant model systems to assess the actual specific contributions of potential growth promotion traits, in order to challenge traditional views on the mechanisms of plant tolerance stimulation. A simple mixture of four volatile metabolites was produced from PsJN, which was capable of mimicking the main effects of bacterial inoculation relative to growth stimulation and tolerance to salinity. To the best of our knowledge, this is the first functional study comparing the effects of volatile compounds produced by *P. phytofirmans* PsJN to other possible growth promotion mechanisms, and reporting their role in plant tolerance to abiotic stress.

## Author Contributions

TL conceived and coordinated the study, performed preliminary experiments and revised the manuscript. SR performed most of the plant inoculation experiments, and contributed to their design and analysis. TT and IP designed and performed specific experiments, related to VOC production and evaluation of PsJN mutants, respectively. MP contributed to the design and analysis of various experiments, supervised part of the work and critically reviewed the manuscript. TG performed analytical detection of salts within tissues and volatile compounds, contributing to the design of related experiments, while PR provided analytical insight on GC-MS experiments, advised on their performance and proper execution, and helped in the interpretation of results. JT and RD performed long term-growth analyses and produced PsJN mutant strains, respectively. All authors contributed to the writing of specific sections of the manuscript and have read and approved its final version.

## Conflict of Interest Statement

The authors declare that the research was conducted in the absence of any commercial or financial relationships that could be construed as a potential conflict of interest.
