# Supplementary material for: Volatile-Mediated Effects Predominate in Paraburkholderia phytofirmans Growth Promotion and Salt Stress Tolerance of Arabidopsis thaliana
Source: Front Microbiol. 2016 Nov 17;7:1838. doi: 10.3389/fmicb.2016.01838 (PMC5112238; doi:10.3389/fmicb.2016.01838)
Supplement: Supplementary file 1 [file Image_1.PDF]

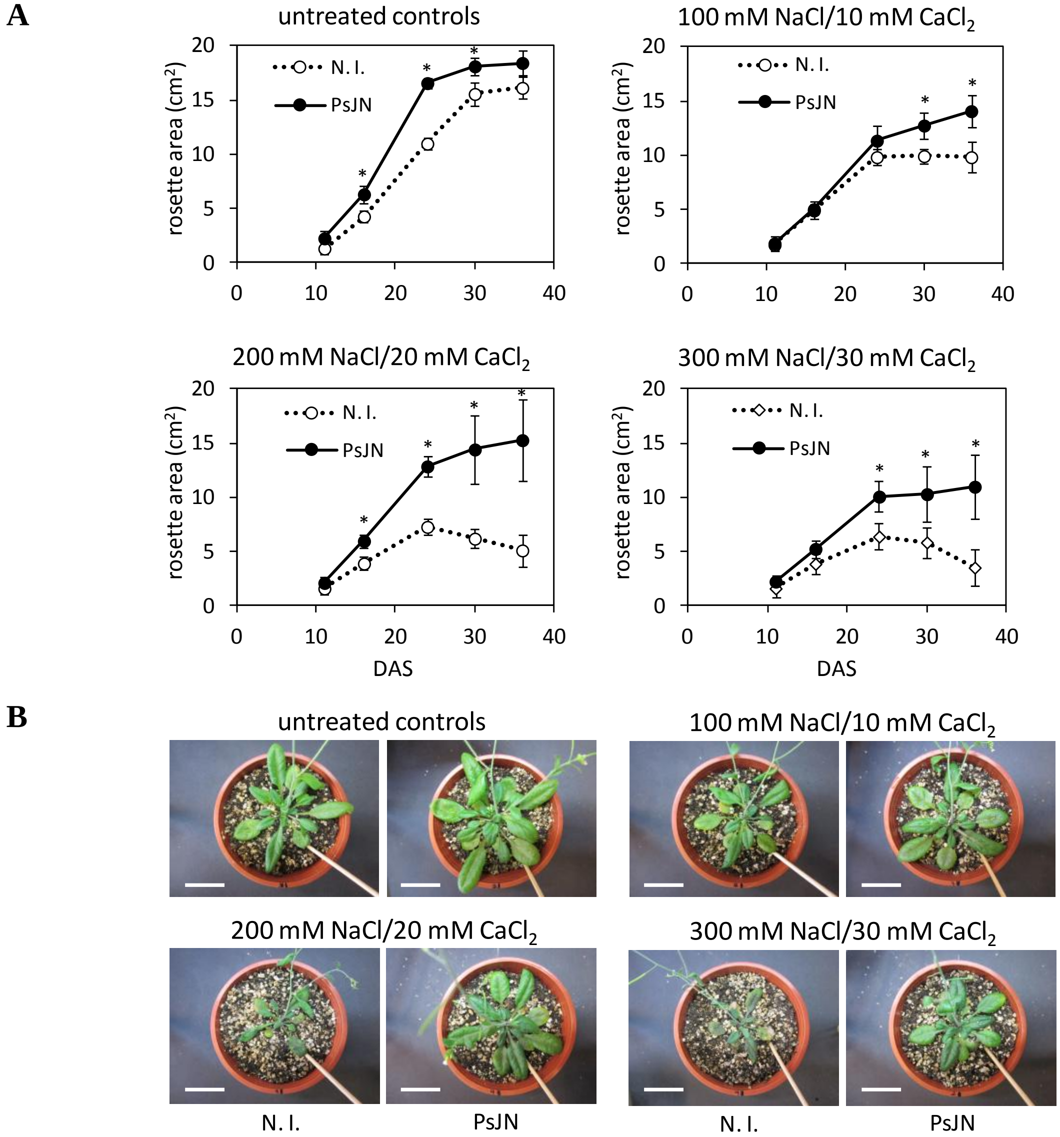

Supplementary Figure S1: Effect of *P. phytofirmans* PsJN inoculation on the growth of *A. thaliana* plants irrigated with different salt concentrations. (A) Changes in rosette area of *A. thaliana* plants inoculated with *P. phytofirmans* (PsJN) and in the absence of bacterial inoculum (Non-inoculated: N. I.). Plants were germinated *in vitro* on half strength MS medium supplemented or not with the bacterium ($1x10^4$ CFU/ml), and transferred at 11 DAS to 1:1 peat:vermiculite substrate. Transferred plants were irrigated 3 times a week. Untreated controls received only standard irrigation water, while treated plants received 2 irrigations with water and a third using different concentrations of NaCl and $CaCl_2$, throughout the week. Asterisks indicate statistically significant differences among PsJN and N. I: plants within each measured time point, which were explored using One-Way ANOVA Tukey's HSD tests; $p < 0.05$. (B) Images of representative plants of each culture condition, taken at 36 DAS.
