# Supplementary material for: Volatile-Mediated Effects Predominate in Paraburkholderia phytofirmans Growth Promotion and Salt Stress Tolerance of Arabidopsis thaliana
Source: Front Microbiol. 2016 Nov 17;7:1838. doi: 10.3389/fmicb.2016.01838 (PMC5112238; doi:10.3389/fmicb.2016.01838)
Supplement: Supplementary file 2 [file Image_2.PDF]

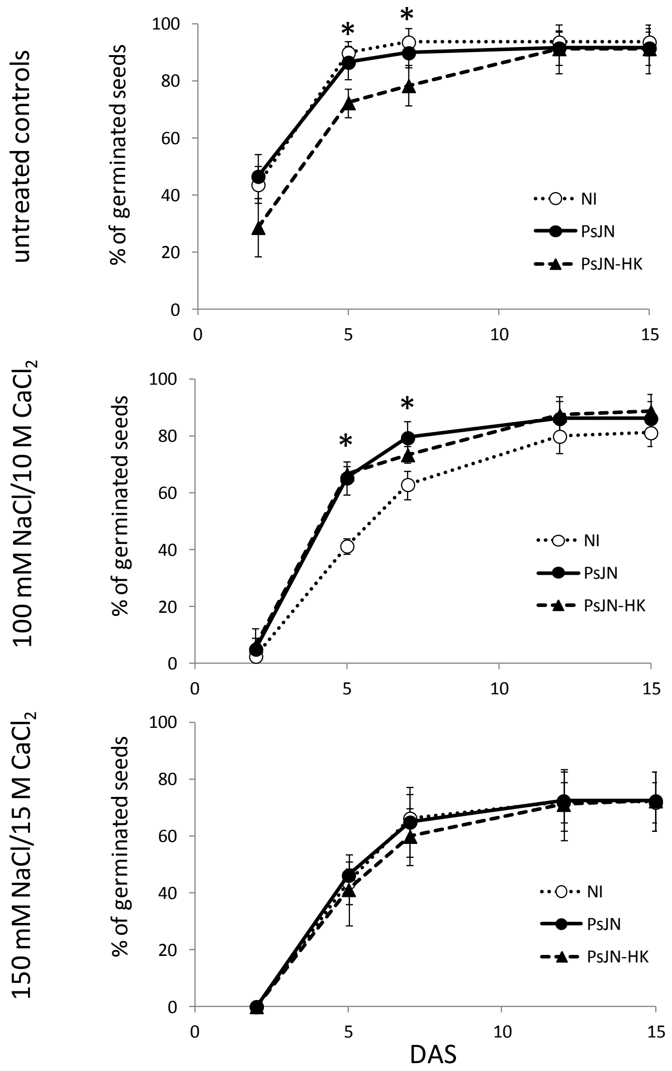

Supplementary figure S2. Effect of *P. phytofirmans* PsJN inoculation on germination of *A. thaliana* plants sown in different salt concentrations *in vitro*. Germination of *Arabidopsis thaliana* col-0 seeds sown in gnotobiotic *in vitro* cultures using half strength MS agar medium inoculated with 1x10^4 CFU/ml of *P. phytofirmans* (PsJN), with 1x10^4 CFU/ml of a heat-killed PsJN inoculum (HK-PsJN), or non inoculated medium (N. I.). Germination was registered visually as the presence of a >3 mm radicule and both cotyledons at different times after sowing for PsJN and N. I. plants grown in standard medium with no added NaCl or $CaCl_2$ (0 mM NaCl/$CaCl_2$) (upper), 100/10 mM NaCl/$CaCl_2$ (middle), or 150/15 mM NaCl/$CaCl_2$ (lower panel). At each time point, the mean percentage values relative to N. I. plants is shown, and the error bars indicate standard deviations from experiments with 80 plants analyzed for each bacterial inoculum and salt treatment. Asterisks indicate statistically significant differences among bacterial treatments within each salt concentration and each measured time point (One way ANOVA Tukey's HSD tests; $p <$ 0.05).
