# Supplementary material for: Volatile-Mediated Effects Predominate in Paraburkholderia phytofirmans Growth Promotion and Salt Stress Tolerance of Arabidopsis thaliana
Source: Front Microbiol. 2016 Nov 17;7:1838. doi: 10.3389/fmicb.2016.01838 (PMC5112238; doi:10.3389/fmicb.2016.01838)
Supplement: Supplementary file 3 [file Image_3.PDF]

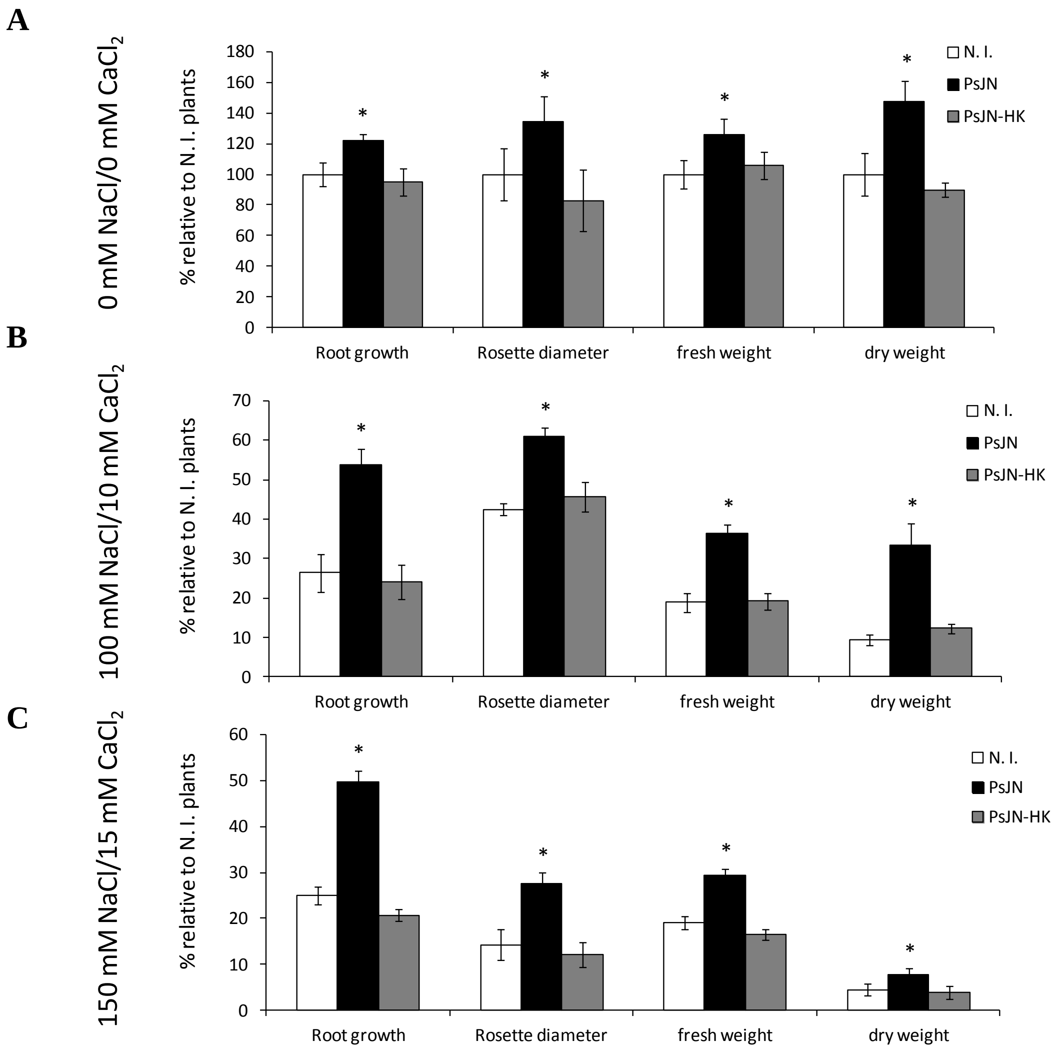

Supplementary figure S3. Effect of *P. phytofirmans* PsJN inoculation on growth of *A. thaliana* plants sown in different salt concentrations *in vitro*. Root growth, rosette diameter and fresh weight of *Arabidopsis thaliana* col-0 grown in gnotobiotic *in vitro* cultures using half strength MS agar medium inoculated with $1x10^4$ CFU/ml of *P. phytofirmans* (PsJN), with $1x10^4$ CFU/ml of a heat-killed PsJN inoculum (HK-PsJN), or non inoculated medium (N. I.). Growth parameters were registered at 21 DAS for PsJN and N. I. plants grown in standard medium with no added NaCl or $CaCl_2$ (0 mM NaCl/$CaCl_2$) (A), 100/10 mM NaCl/$CaCl_2$ (B), or 150/15 mM NaCl/$CaCl_2$ (C). Bars show mean percentage values relative to N. I. plants, and the error bars indicate standard deviations from experiments with 30 plants analyzed for each bacterium and salt treatment. Asterisks indicate statistically significant differences among bacterial treatments for each separate parameter within each salt concentration (One way ANOVA Tukey's HSD tests; $p < 0.05$).
