# Supplementary material for: Volatile-Mediated Effects Predominate in Paraburkholderia phytofirmans Growth Promotion and Salt Stress Tolerance of Arabidopsis thaliana
Source: Front Microbiol. 2016 Nov 17;7:1838. doi: 10.3389/fmicb.2016.01838 (PMC5112238; doi:10.3389/fmicb.2016.01838)
Supplement: Supplementary file 4 [file Image_4.PDF]

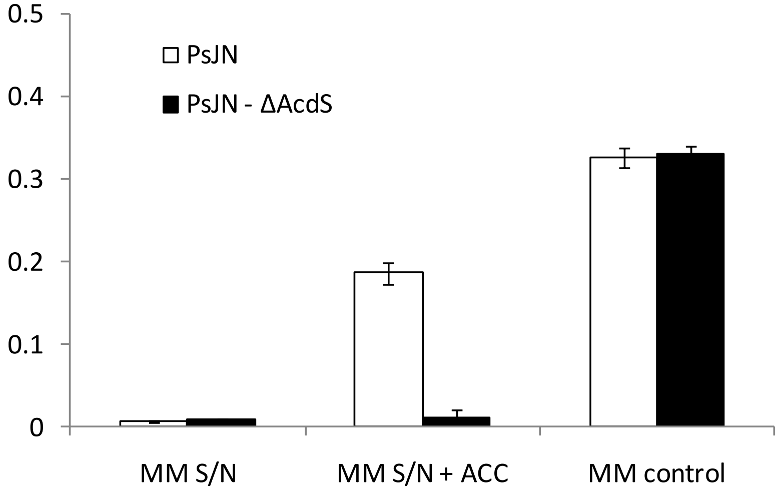

Supplementary figure S4. Growth assay on ACC as a sole nitrogen source for *Paraburkholderia phytofirmans* PsJN-*acdS* mutant. Growth of the wild type *P. phytofirmans* PsJN (PsJN) or the *P. phytofirmans* PsJN-*acdS* mutant (PsJN-ΔAcdS) on ACC as a sole nitrogen source was evaluated using a modified version of liquid Dorn (Dorn *et al*., 1974) minimal medium without added nitrogen (MM S/N), the same medium supplemented with 2 mM ACC (MM S/N+ACC), or standard Dorn medium containing nitrogen sources (MM Control). O.D. 600 of each culture was measured after 72 h of incubation at 30ºC in agitation. Results are means of three replicate cultures for each inoculum and medium.
