# Supplementary material for: Volatile-Mediated Effects Predominate in Paraburkholderia phytofirmans Growth Promotion and Salt Stress Tolerance of Arabidopsis thaliana
Source: Front Microbiol. 2016 Nov 17;7:1838. doi: 10.3389/fmicb.2016.01838 (PMC5112238; doi:10.3389/fmicb.2016.01838)
Supplement: Supplementary file 5 [file Image_5.PDF]

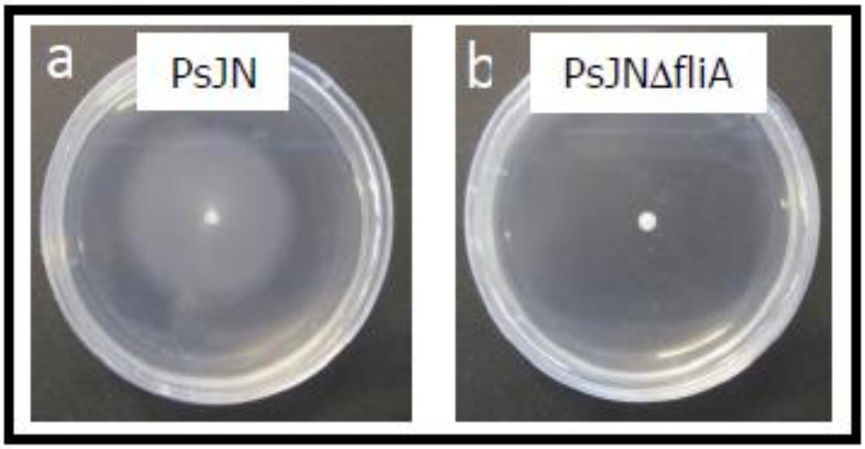

Supplementary figure S5. Swimming motility assay for *Paraburkholderia phytofirmans* PsJN-*FliA* mutant. Swimming motility was evaluated by inoculation of wild type *P. phytofirmans* PsJN (a) or the *P. phytofirmans* PsJN-*FliA* mutant (b), in the center of a minimal medium 0.25% agar plate, supplemented with 10 mM fructose. Motility is evidenced as the advance of the bacterial front when the substrate is consumed at the site of inoculation. Plates were photographed after 5 days incubation at 30ºC.
