# Supplementary material for: Volatile-Mediated Effects Predominate in Paraburkholderia phytofirmans Growth Promotion and Salt Stress Tolerance of Arabidopsis thaliana
Source: Front Microbiol. 2016 Nov 17;7:1838. doi: 10.3389/fmicb.2016.01838 (PMC5112238; doi:10.3389/fmicb.2016.01838)
Supplement: Supplementary file 6 [file Image_6.PDF]

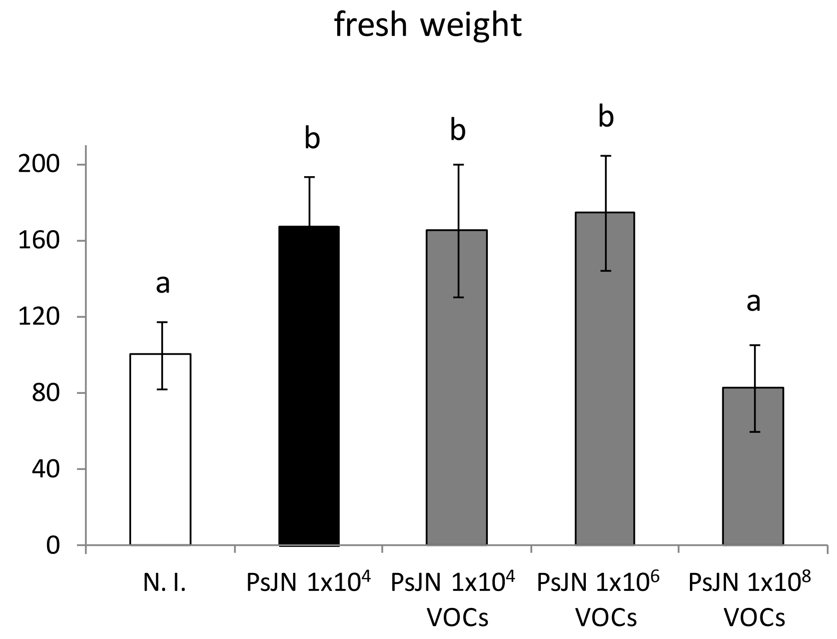

Supplementary figure S6. Effect of bacterial inoculum concentration in MS$^1/_2$ agar on the growth of *A. thaliana* plants co-incubated with bacteria in dual plate systems. Fresh weight of *Arabidopsis thaliana* col-0 grown in gnotobiotic *in vitro* cultures grown in half strength MS agar medium directly inoculated with *P. phytofirmans* (PsJN), at 1x10^4 CFU/ml (black bar), grown in agar placed in front of MS$^1/_2$ inoculated with 1x10^4, 1x10^6, or 1x10^8 CFU/ml (gray bars), or non inoculated medium (N. I.; white bar). Growth parameters were registered at 21 DAS for all plants. Bars show mean percentage values relative to N. I. plants, and the error bars indicate standard deviations from experiments with 24 plants analyzed for each inoculums type. Letters indicate statistically significant differences among bacterial treatments for each separate parameter within each salt concentration (One way ANOVA Tukey's HSD tests; $p < 0.05$).
