# Supplementary material for: Volatile-Mediated Effects Predominate in Paraburkholderia phytofirmans Growth Promotion and Salt Stress Tolerance of Arabidopsis thaliana
Source: Front Microbiol. 2016 Nov 17;7:1838. doi: 10.3389/fmicb.2016.01838 (PMC5112238; doi:10.3389/fmicb.2016.01838)
Supplement: Supplementary file 7 [file Image_7.PDF]

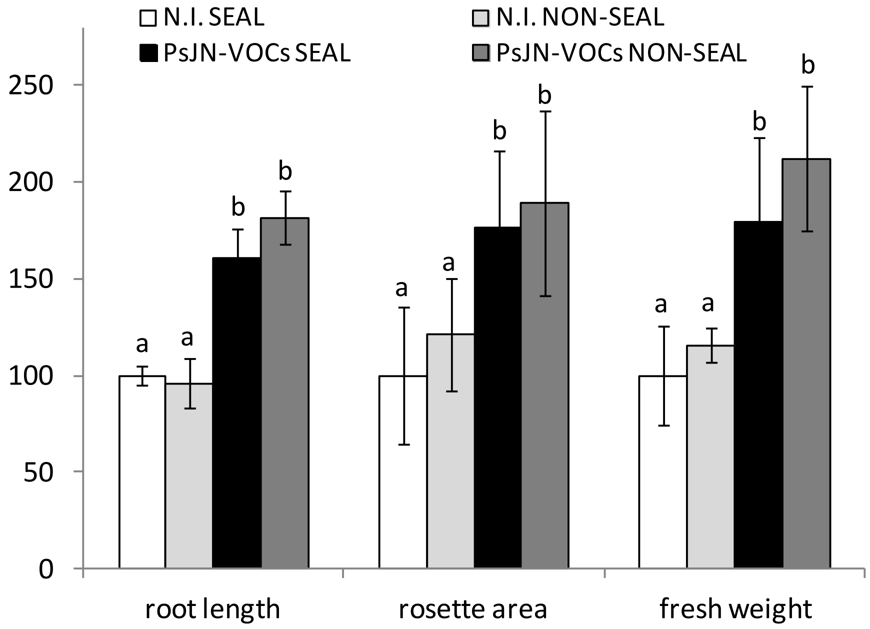

Supplementary figure S7. *A. thaliana* growth stimulation by volatile compounds emitted by *P. phytofirmans* PsJN on sealed and unsealed systems. Root length, rosette area and fresh weight were determined for *Arabidopsis thaliana* col-0 grown *in vitro* in MS$^{1}/_{2}$ agar medium placed in front of MS$^{1}/_{2}$ inoculated with $1x10^{4}$ CFU/ml of strain PsJN (PsJN-VOCs), or non inoculated medium (N. I). Results are compared among parafilm sealed petri dishes (PsJN-VOCs SEAL or N.I. SEAL), and replicate systems in the absence sealing (PsJN-VOCs NON-SEAL; N.I. NON-SEAL). Growth parameters were registered at 21 DAS. Columns show mean values, and the error bars indicate standard deviations from experiments with 24 plants analyzed for each compound concentration. Asterisks indicate statistically significant differences from the control (N. I.) treatment within each compound (One way ANOVA Tukey's HSD tests; $p < 0.05$).
