# Supplementary material for: Volatile-Mediated Effects Predominate in Paraburkholderia phytofirmans Growth Promotion and Salt Stress Tolerance of Arabidopsis thaliana
Source: Front Microbiol. 2016 Nov 17;7:1838. doi: 10.3389/fmicb.2016.01838 (PMC5112238; doi:10.3389/fmicb.2016.01838)
Supplement: Supplementary file 8 [file Image_8.PDF]

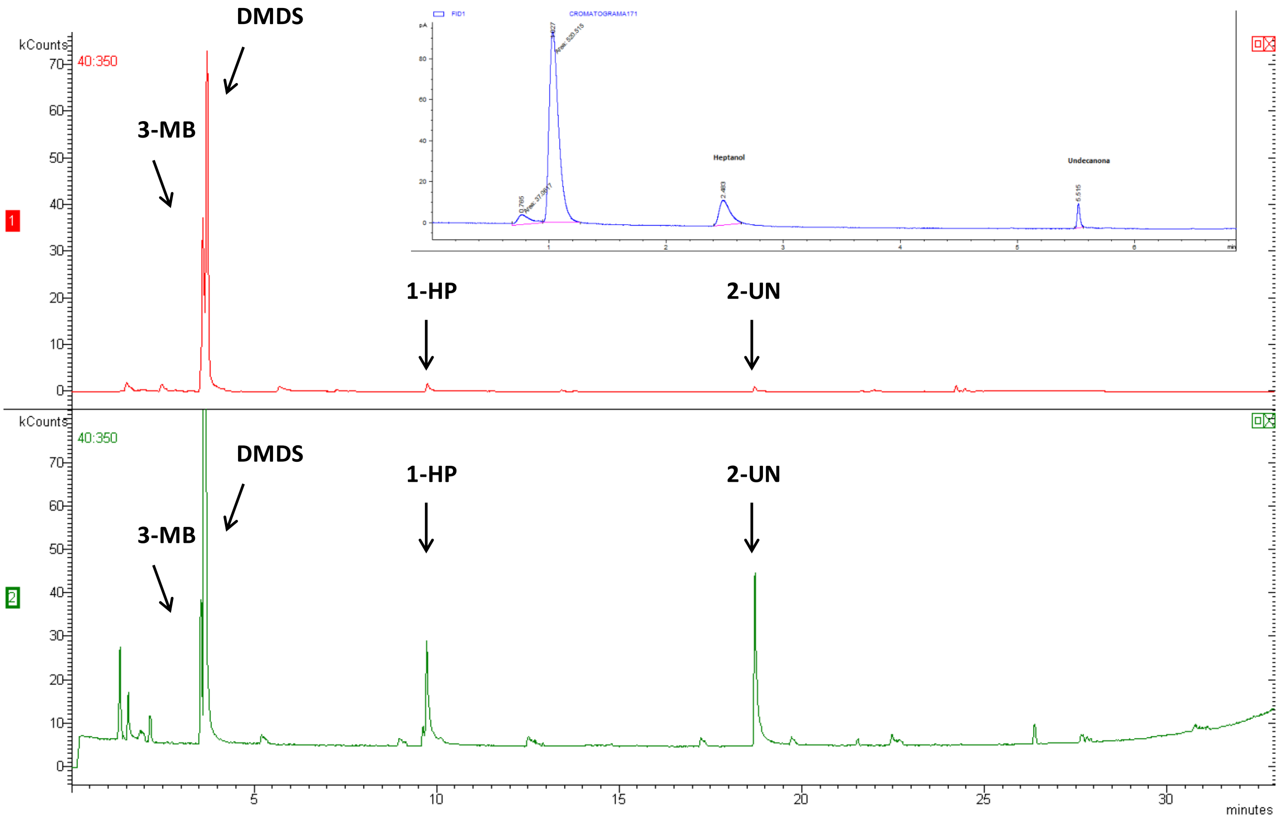

Supplementary figure S8. Gas chromatogram of *P. phytofirmans* PsJN volatile emission. Volatile emissions captured from the headspace of *P. phytofirmans* cultures grown in King's B agar medium using a SPME fiber as described in materials and methods (above). Volatile emissions from the headspace of a stock mixture made of equal liquid volumes of pure 3-MB, DMDS, 1-HP and 2-UN (below). Retention times of stock compounds were 3.55 min (3-MB); 3.70 min (DMDS); 9.73 min (1-HP); and 18.72 min (2-UN), which are identical to retention times for signals in the sample chromatogram. Results are shown at the same scale, comparing the complete GC profile. MS fragmentation profiles are identical among sample and standard peaks, and were all confirmed by the NIST library. Small additional peaks in the sample chromatogram correspond to siloxane-derived compounds released by the fiber and/or the GC column. Inset shows a GC chromatogram of King's B grown *P. phytofirmans* emissions, obtained by direct headspace analysis and the respective compounds detected by FID.
