# Supplementary material for: Volatile-Mediated Effects Predominate in Paraburkholderia phytofirmans Growth Promotion and Salt Stress Tolerance of Arabidopsis thaliana
Source: Front Microbiol. 2016 Nov 17;7:1838. doi: 10.3389/fmicb.2016.01838 (PMC5112238; doi:10.3389/fmicb.2016.01838)
Supplement: Supplementary file 9 [file Table_1.PDF]

Supplementary Table 1: Fresh weight, dry weight and percentage of water content in *Arabidopsis thaliana* plants irrigated with salt.

	Salt in irrigation water					
	0/0 mM NaCl/$CaCl_2$ (control)			200/20 mM NaCl/$CaCl_2$		
	FW (mg)	DW (mg)	% of water*	FW (mg)	DW (mg)	% of water
N. I.	403 ± 47^{a}	97 ± 21^{a}	76.2 ± 1.4	80 ± 18^{a}	50 ± 9^{a}	37.1 ± 4.9^{a}
PsJN	531 ± 30^{b}	133 ± 19^{b}	75.1 ± 2.4	259 ± 28^{b}	99 ± 11^{b}	61.9 ± 0.5^{b}
PsJN-HK	321 ± 36^{c}	89 ± 16^{a}	73.7 ± 2.6	91 ± 19^{a}	53 ± 10^{a}	42.1 ± 8.1^{a}

Letters indicate significant statistical differences within each column, analyzed by one-way ANOVA Tukey test with $p<0.05$.

*No significant statistic difference could be detected for the % of water of control plants with different bacterial inocula.
