# Supplementary material for: Volatile-Mediated Effects Predominate in Paraburkholderia phytofirmans Growth Promotion and Salt Stress Tolerance of Arabidopsis thaliana
Source: Front Microbiol. 2016 Nov 17;7:1838. doi: 10.3389/fmicb.2016.01838 (PMC5112238; doi:10.3389/fmicb.2016.01838)
Supplement: Supplementary file 10 [file Table_2.PDF]

Supplementary Table 2: Sodium ion concentrations per mg of dry tissue weight in inoculated *Arabidopsis thaliana* plants grown *in vitro* in the presence of salt

$NaCl/CaCl_2$ Concentration (mM)	**rosette**			**root**		
	N. I.	PsJN	PsJN-HK	N. I.	PsJN	PsJN-HK
0	12.8 ± 1.7^{a}	8.4 ± 1.6^{a}	13.1 ± 2.9^{a}	5.56 ± 0.9^{a}	6.1 ± 0.8^{a}	8.1 ± 3.7^{a}
100/10	78.9 ± 6.0^{a}	60.3 ± 5.1^{b}	84.8 ± 7.9^{c}	45.5 ± 7.0^{a}	53.2 ±19.2^{a}	49.7 ± 4.7^{a}
150/15	131.7 ± 25.2^{a}	86.2 ± 19.0^{b}	224.9 ± 66.2^{c}	62.4 ± 10.0^{a}	74.10 ± 13.0^{a}	68.1 ± 11.8^{a}

Sodium concentrations are expressed as mg of Na^{+}/g of DW. Letters indicate significant statistical differences within each row and tissue, analyzed by one-way ANOVA Tukey test with $p<0.05$.
